# The High-Resolution Archaeology of Shared Courtyards at Old Dongola (14th–16th Century a.d., Sudan): an Intensive Approach to Domestic Open Spaces

**DOI:** 10.1080/00934690.2024.2397883

**Published:** 2024-09-12

**Authors:** Maciej Wyżgoł, Mohammed Nasreldein, Agnieszka Ryś-Jarmużek

**Affiliations:** 1 University of Warsaw, Warsaw, Poland; 2 University of Tübingen, Tübingen, Germany; 3 University of Gezira, Wad Madani, Sudan

**Keywords:** Open spaces, household, high-resolution archaeology, multi-elemental analysis, spatial statistics

## Abstract

Identifying the dynamics of domestic open spaces remains a challenging task. This research applies an adjusted theoretical framework of activity areas to characterize domestic open spaces in the 14th–16th century a.d. in Old Dongola, Sudan. Activity areas were defined as sedimentations of residues of recurring cycles of changing actions rather than stable components of space. To identify domestic space, this research utilizes high-resolution methods: analyses of multiple chemical elements, spatial distribution of objects, and botanical remains of courtyard occupational surfaces, combined with spatial statistics using local Moran’s I autocorrelation. The relationships between the remains of human and non-human actions are discussed in terms of the material affordances affecting their deposition within the archaeological layers. Application of these methods allowed for the identification of areas of domestic tasks related to high concentrations of elements, as well as clusters of tools located on their edges. Botanical data corroborated often vague identifications of activities based on geochemistry.

## Introduction

In this article, we present the results of an activity area analysis, based on the spatial analysis of elements, artifacts, and botanical remains, within the occupation layers of shared courtyards from two consecutive house compounds dating to the 14th–16th centuries a.d. in the city of Old Dongola, northern Sudan. The main objective was to combine different datasets to determine small-scale activity areas within these large, open, and mostly empty spaces in order to provide a more nuanced narrative of the use of domestic space through the action of house dwellers.

Understanding the dynamics of urban open spaces (Jervis et al. 2021), whether they are public squares, streets, or domestic courtyards, is crucial in comprehending their purpose and the way in which these ambiguous spaces developed. In a domestic context, courtyards served as spaces where house dwellers interacted and consequently negotiated their identities and social status. In many African societies, such domestic spaces played an important part in the social life of city dwellers (Steyn 2005). In the absence of open squares, house courtyards sometimes adopt some of a public square’s functions, e.g., such as trade on the Swahili coast (Wynne-Jones 2013; Baumanova 2022). Such public open spaces were also lacking in Old Dongola, so house courtyards played a central role not only in households but in the city as a whole. They acted as a gathering point for house occupants and their visitors, as well as a domestic workspace (Wyżgoł and Deptuła 2023, 13–17). However, in Old Dongola, these courtyards have often been under-investigated and labeled with the vague term “multifunctional” (Obłuski, Dzierzbicka, and Maślak 2021, 240–244; Wyżgoł 2021a), which generally reflects the limitations of the archaeological evidence associated with them and, consequently, their archaeological narratives. Identifying activity areas within Dongolese courtyards poses a major research challenge due to the unpredictable nature of site formation processes and the varying treatment of specific spaces in terms of cleaning and waste deposition, as well as the high mobility of the associated material culture (Obłuski and Dzierzbicka 2022).

The investigation of activity areas—defined as a specific location where raw materials, products, and artifacts accumulate as a consequence of human activities (Pfälzner 2015, 33–40)—can be significantly improved through high resolution analysis of archaeological materials and their spatial associations (Raja and Sindbæk 2020). Among these approaches, multi-elemental analysis (Middleton and Price 1996; Wells 2004; Milek and Roberts 2013; Sulas, Kristiansen, and Wynne-Jones 2019; Dalton 2021; Wyżgoł and Woronko 2024) holds the potential to map both natural processes, such as soil erosion/deposition, and human-generated contributions, such as food processing and manufacturing. Thus, it can successfully complement archaeobotanical and artifact distribution analysis.

The aims of this paper were to assess the utility of combining analyses of elemental composition of floors and the distribution of artifacts and botanical remains in the reconstruction of small-scale activity areas within a domestic context at this specific archaeological site in the Middle Nile Valley. Additionally, it examines the spatial relationships between these types of residues. The study also aims to apply high resolution archaeological methodologies (Raja and Sindbæk 2020) to approach an African city’s domestic courtyards in intensive terms (see Jervis et al. 2021), challenging the generalizing term “multifunctionality.”

## Material and Methods

### Old Dongola: the city and its households

**Figure 1.**
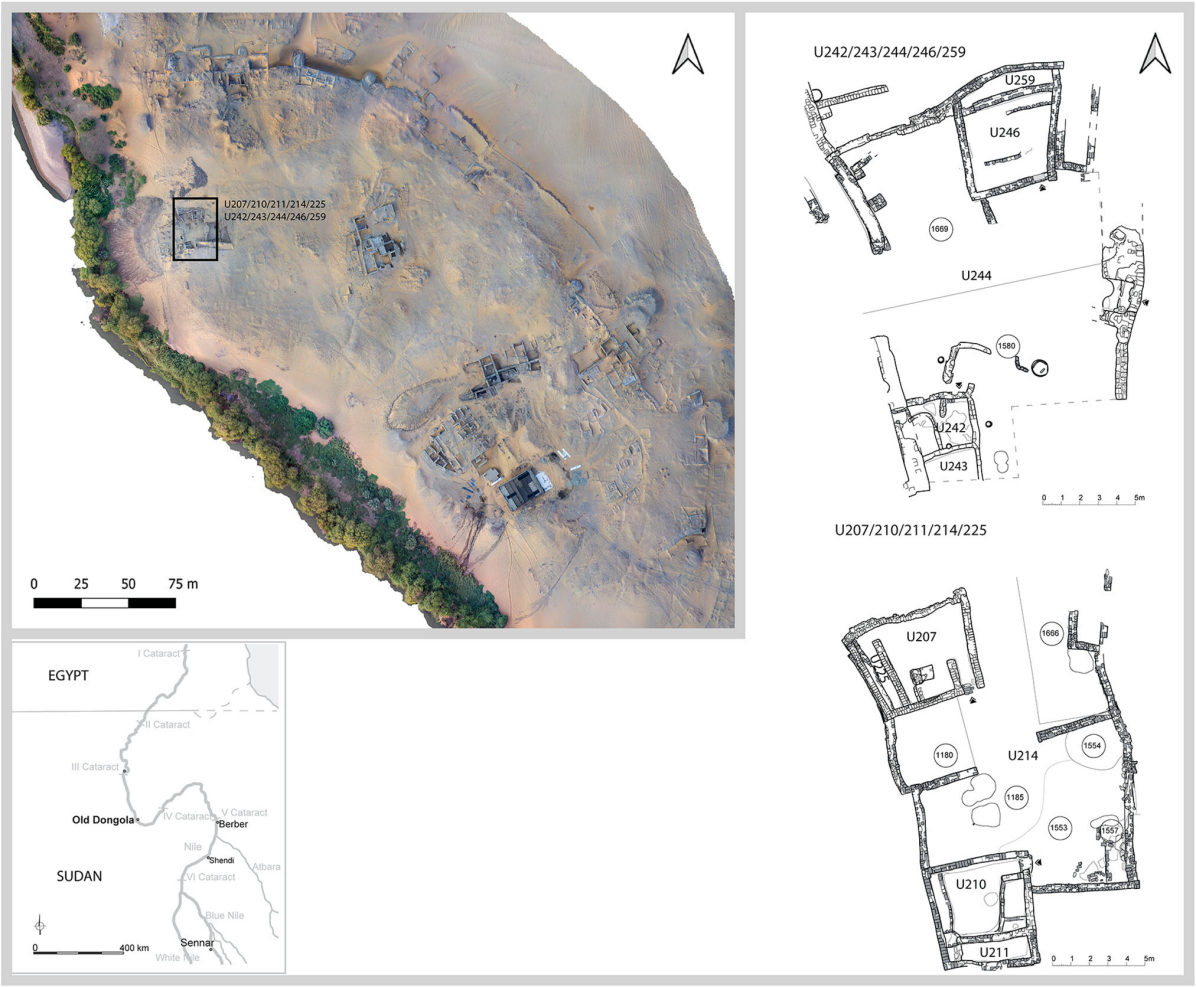
A map of the Middle Nile Valley and orthophoto of the archaeological site in Old Dongola with the marked location of the studied house compound and plans of the excavated house compounds with indicated numbers of analyzed spaces (U …) and depositions (Arabic numerals inscribed in circles) (drawing by A. Wujec and D. Zielińska; photo by A. Chlebowski).

Old Dongola (Figure 1) is situated in contemporary Sudan between the Third and Fourth Nile Cataracts. From the 6th–14th century a.d., it was the capital of the Christian Kingdom of Makuria. Following the decline of Makuria, Old Dongola later became the capital of a small polity known as the Kingdom of Dongola, and from the 16th century a.d., it then fell under the Funj Sultanate sphere of influence. The city sustained its significant political and economic significance in the Middle Nile Valley until the 19th century a.d., when the Turco-Egyptian forces of Muhammad Ali conquered the Funj Sultanate. During the existence of the Kingdom of Dongola, after the fall of Makuria (14th–15th century a.d.), and during the domination of the Funj Sultanate (16th–19th century a.d.), clusters of residential compounds comprised the city separated by an irregular network of streets within and outside the city walls (Obłuski, Herbich, and Ryndziewicz 2022). The walled city center during the Funj period was nearly unrecognizable from the rest of the settlement and was inhabited by both commoners and the aristocracy (Obłuski, Dzierzbicka, and Maślak 2021).

In the latest contexts associated with the Kingdom of Makuria, and later in the Kingdom of Dongola, residential areas in Old Dongola were characterized by enclosed spaces composed of more than one building, which were identified as a single residential unit, along with a shared courtyard. The houses consisted of a residential unit that was usually equipped with an additional narrow storage room located behind it and, in some instances, vestibules (de Lellis, Maślak, and Wyżgoł 2021, 86–95; Wyżgoł 2021b, 41–50; 2021c, 187–193; Wyżgoł and Deptuła 2023). The interior of residential units was swept, removing most of the anthropogenic macro-residues. Furthermore, a limited set of installations including benches only attest to activities with a very low residuality, which was confirmed by geochemical analysis of the floors (Wyżgoł and Woronko 2024). Some compounds had separate buildings that could be interpreted as kitchens, equipped with quern emplacements and hearths (Wyżgoł 2021b, 45; 2021d, 193). The courtyards, which provided access to every house within a compound, were shared by their residents. Consequently, they constituted spaces where most interactions between the house dwellers occurred. The usage of such open spaces is often attested by installations like quern emplacements, hearths, and clay storage bins. The composition of occupational deposits further suggests that animals freely roamed in these areas. Although the equipment found in a courtyard varied from one compound to another, some did not have any built-in features (de Lellis, Maślak, and Wyżgoł 2021, 95; Deptuła 2021, 208; Maślak and Deptuła 2021, 136). The house inheritance pattern represented by subsequent abandonments and re-inhabitations of houses within the compounds suggest that single houses were inhabited by nuclear families (Wyżgoł forthcoming).

The two courtyards discussed in this article (see Figure 1) belonged to two spatially overlapping house compounds built consecutively, separated only by a period of abandonment. The earlier house compound, U242/243/244/246/259, was comprised of at least two buildings. A two-room house, U246/259, had the most typical arrangement as observed from the 14th–18th century a.d. in Old Dongola, comprising a living room and a narrow storage space. U242/243 was a separate building with two rooms and independent entrances. Heavily eroded walls to the north of U242 suggest that another structure might have been situated there. The shared courtyard U244 was entered from the east and was furnished with three clay containers and a pit in its southern part. This house compound was fully excavated to the north, east, and west, while the southernmost part was not excavated. The life cycle of this house compound lasted approximately 50 years from the mid-14th to the beginning of the 15th century a.d. (Table 1). The examined occupational depositions of the courtyard were dated to the second half of the 14th century a.d. While Building U246/259 was a house, representing the dominant type like U246/259, most likely inhabited by a nuclear family (Wyżgoł and Deptuła 2023; Wyżgoł forthcoming), the interpretation of the function of Building U242/243 is more uncertain. Numerous constructions with a similar layout are attested in different parts of the city in the 13th–14th century a.d. Based on the equipment—a bench and a stub wall—the structure is probably a dwelling (Dzierzbicka and Deptuła 2018; Wyżgoł forthcoming), although it differed significantly from typical house architecture. Other possible interpretations as a domestic workspace are not supported by any installations such as hearths or querns.

**Table 1.** Radiocarbon dates of samples collected from both house compounds.

House compound	U242/243/244/246/259	U207/210/211/214/225
cal a.d.	1366–1392 (95.4%)	1468–1522 (95.4%)

After the abandonment of this compound, another multiphase compound was constructed in the same location. This was continuously inhabited for approximately 200 years, from the mid-15th to the end of the 17th century a.d. The analyzed occupational depositions from the courtyard are dated from the second half of the 15th to the first half of the 16th century a.d. (see Table 1). The number and location of houses within the compound underwent multiple changes. In the stage contemporary with the analyzed floors, this compound included U207/210/211/214/225 and was comprised of two two-room houses with narrow storage spaces: U207/225 and U210/211. A shared courtyard, U214, was reached from the north and partially separated by two parallel walls in the middle, leaving a passage between them. A hearth was in the southeastern corner of the northern part, and another two were in the southeastern corner of the southern part with a presumed quern emplacement adjacent. Two pits were located in the central-western part. Two houses, representing typical house architecture for this period (Obłuski, Dzierzbicka, and Maślak 2021), were most likely inhabited by two related nuclear families. House U210/211 was the first house built in the compound in the second half of the 15th century a.d. It is possible that during the analyzed phase, at least 20 years later, it was still inhabited by the founders of the compound, while U207/225, built at a later architectural phase, was inhabited by a younger generation (Wyżgoł forthcoming).

Occupational deposits in both courtyards consisted primarily of accumulated aeolian sand with significant admixture of organic matter, mainly plant remains and animal dung, as well as Nile silt from eroding walls surrounding both courtyards. The proportions within these deposits were uneven across the surfaces. Additionally, significant inclusions of ashes and pottery fragments, as well as some animal bones and a few artifacts, were observed in the deposits of both courtyards (Table 2).

**Table 2.** Relative quantity of animal bones and pottery (from + to +++), as well as types and number of household utensils found in the occupational depositions in the courtyards.

Courtyard	Deposition no.	Animal bones	Pottery	Household utensils
U214	1180	+	++	
	1185	+	++	spindle whorl (1)
	1553	+	++	fire dog (1)
	1554			
	1557		+	
	1666	+	++	fire dog (2)
U244	1580	+	++	
	1669	+	+	spindle whorl (1)

### Analysis of activities in domestic courtyards

The use of high-resolution archaeological methods (Raja and Sindbæk 2020) allowed us to reassess seemingly empty urban spaces (Jervis et al. 2021) such as squares and courtyards. These methods provide the data to allow us to study courtyards as intensive spaces, which are defined by the performance of household activities, as well as social and material encounters, eventually leading to the emergence of household communities (Jervis 2016). This approach challenges the idea of space being defined predominantly by physical structures and boundaries, in which paradigm courtyards have often been understood as empty spaces (DeLanda 2016, 76). It also allows for the construction of intricate occupational histories (Wandsnider 2008) of the open spaces. Archaeologically detected human-related activity areas provide snapshots of the movement of objects through this domestic space, allowing for an observation of the relationships that emerged during the performance of household tasks. The analysis of the activity areas employed in this study derives from a conceptual framework explained by Pfälzner (2015). Its main assumption is that archaeologically identified residues are the sum of different activities carried out in a specific part of the space in the past. However, it is important to note that the interpretative strength of the evidence can vary depending on the type of residue (Pfälzner 2015, 36–41). Moveable objects such as tools, containers, pottery, and archaeozoological and archaeobotanical remains were considered weaker indicators of activities, as these can be moved around easily. The mobility of items such as processing tools and the preservation bias of material such as different plant parts may affect the differentiation between activity area and place of storage and/or disposal of residues. Nonetheless, such finds can still be useful in the reconstruction of a broader activity zone. Fixed installations such as hearths or quern emplacements are considered more reliable indicators of activities in specific areas.

This framework has some constraints, particularly as open areas are affected by changing weather conditions and seasonal and daily alterations. Therefore, in this study we assume that, rather than individual tasks (static in both time and space performed in structured spaces), the activity areas represent spatially overlapping activities (Merrill and Read 2010). The ethnoarchaeological study by Rondelli and colleagues shows that the archaeological activity areas may represent a sum of cycles of use depending on seasonal change, which occur over several years (Rondelli et al. 2014, 486–489). Furthermore, the higher the level of residuality (i.e., production of sufficient waste material), the more represented activity is visible in the archaeological record (Clark 2017, 1301). This suggests that the pattern of disposal can then be more accessible through analysis of activity areas than the pattern of use (Clark 2017, 1302). Such constraints result in an assumption that activity area analysis has the capacity to provide insight into the overall intensity of the activities, rather than their actual organization occurring at a given time.

Even single spatial analyses of human action residues can offer valuable information about the functioning of households, but their interpretative power can be enhanced when integrated into a multi-method technique (Sulas, Kristiansen, and Wynne-Jones 2019; Dalton 2021; Reid et al. 2023). In this study, we selected specific categories of residues, differing in scale, whose location was recorded in accordance with the excavations’ methodology. After selection, geochemical analysis, investigation of the function and distribution of ground stone tools, and identification of botanical remains were carried out. Faunal remains (Osypińska forthcoming) and pottery (Danys 2022) were collected in bulk from anthropogenic deposits. However, although informative in terms of function of space, they did not provide detailed information on the spatial distribution of household activities.

Each category of domestic remains under analysis was affected by different human-related and/or environmental processes. Elemental residues in this material showed evidence of undergoing chemical reactions, although, due to environmental conditions, this could only be observed at a limited scale (Wyżgoł and Woronko 2024). This encompassed leaching, as well as bonding, creating stable compounds (Oonk, Slomp, and Huisman 2009). Tools were frequently linked to human activity, specifically reuse and ultimate abandonment in either the place of use or middens (Schiffer 1983, 1996; LaMotta and Schiffer 1999). Botanical remains were likely deposited in storage bins and pits, either intentionally or unintentionally, or left as by-products during processing, e.g., dehusking, or discarded in middens. While the ultimate fate of stone tools was determined by their sturdiness, meaning they were either disposed of or reused/relocated, they could also be moved through unintentional human activity and natural geological processes (Simms and Heath 1990; Clark 2017, 1306–1307); the movement of botanical remains was also impacted by environmental factors (Enloe 2006) such as wind. Additionally, composition of botanical remains may have been influenced by small rodents and birds, as they tend to feed on grains found during excavations, as well as decomposed by microbiota.

### Geochemistry

#### Multi-elemental analysis

Multi-elemental analysis was selected as a cost- and time-effective geochemical method to provide dense spatial data of micro-scale residues of human actions. The analyses using Inductively Coupled Plasma-Atomic Emission Spectrometry (ICP-AES) and Inductively Coupled Plasma-Mass Spectrometry (ICP-MS) provide an opportunity to compare datasets from different elements and are sensitive to subtle changes in chemical composition, allowing for the distinction between a wide range of human activities with high precision (Wilson, Davidson, and Cresser 2008, 2009; Middleton et al. 2010; Fleisher and Sulas 2015).

The courtyard spaces were divided into grids with a resolution set to 1  m to create a map of the area and ensure an adequate sampling density. The samples were collected from well-established contemporary surfaces. The export of samples from Sudan was carried out based on consultations with Sudanese authorities and permits obtained from the National Corporation of Antiquities and Museum of the Republic of Sudan. Prior to both analyses, the samples were ground in a ceramic mortar and sieved (0.1  mm). All samples were analyzed at the laboratory of the Bureau Veritas Minerals (Canada). Multi-elemental analysis of the courtyard’s U214 floor was performed by ICP-AES to determine the concentration of 35 elements (Ag, Al, As, Ba, Be, Bi, Ca, Cd, Co, Cr, Cu, Fe, K, La, Mg, Mn, Mo, Na, Nb, Ni, P, Pb, S, Sb, Sc, Sn, Sr, Th, Ti, U, V, W, Y, Zn, and Zr). Due to an error in the communication with the laboratory, analysis of the floor in Courtyard U244 was performed using a more precise method, ICP-MS, determining the concentration of 45 elements (Ag, Al, As, Ba, Be, Bi, Ca, Cd, Ce, Co, Cr, Cu, Fe, Hf, In, K, La, Li, Mg, Mn, Mo, Na, Nb, Ni, P, Pb, Rb, Re, S, Sb, Sc, Se, Sn, Sr, Ta, Te, Th, Ti, Tl, U, V, W, Y, Zn, and Zr). The analyses used a strong multi-acid extraction. This entailed heating samples in HNO_3_, HClO₄, and HF to fuming and taking them to dryness; the residue was then dissolved in HCl before analysis. In order to reliably compare both datasets, results of the ICP-MS analysis with values lower than the level of detection in the ICP-AES analysis were discarded, and additional elements not included in ICP-AES were not taken into consideration. The values of elements in floor samples were compared to the background level. In this study, the background level was calculated as the mean of sample values taken from sun-dried bricks, which is closest to the main building material of the analyzed floors (see Wyżgoł and Woronko 2024).

#### Statistics and spatial statistics

The distribution of elemental values was mapped using QGIS software. In order to reinforce the interpretations and to mitigate any effect of past anthropogenic inputs already present in the building material (see Santiago-Marrero, Lancelotti, and Madella 2024, 4), Moran’s I and Local Moran’s I spatial autocorrelation (Moran 1950) were calculated to identify non-random distribution of high and low values of elements. In the case of Moran’s I, p > 0.05, implying random distribution of high and low values, Local Moran’s I was not calculated. Significant clustering of high and low values (p < 0.05) was determined by calculating Local Moran’s I and was plotted on maps. Both calculations were performed using the Spatial Analysis Toolbox plugin in QGIS (Delialis 2022).

### Archaeobotanical analysis

A methodical archaeobotanical sampling strategy was conducted in order to determine spatial variations in the distribution of plant remains. We collected 10–20 L of sediment samples from Courtyards U214 and U244. This analysis focuses on the results obtained from 17 samples collected from nine archaeological contexts. The sediment samples were collected from different deposits, mostly identified as tamped animal dung, middens, occupational layers, and hearth fills.

During the winter 2022–2023 excavation season, the sediment samples were processed using a bucket flotation method (Pearsall 2015; Champion and Fuller 2018). The buoyant material was poured off and collected in a metal mesh sieve (2  mm, 1  mm, 0.5  mm, and 0.2  mm mesh sizes). The procedure was repeated by adding more water and stirring three to four times for each sample to ensure that all plant remains were floated. A 1  mm mesh size was used for the heavy fractions remaining in the bucket’s bottom. All fractions were labeled and backed in fine nets (0.5  mm) for drying and then packed for transport. The export of samples from Sudan was carried out based on consultation with Sudanese authorities and permits obtained from the National Corporation of Antiquities and Museum of the Republic of Sudan.

The fine floating materials were further processed in the archaeobotanical laboratory of the Institute for Archaeological Sciences, University of Tübingen, Germany. Prior to sorting, each sample was sifted through four nested geological screens with mesh sizes of 2  mm, 1  mm, 0.5  mm, and 0.2  mm, and then each fraction was sorted individually. Seed identification is based on anatomical and morphological comparison with fresh seeds from the reference collection specimens in the archaeobotanical laboratory at the University of Tübingen and seed identification atlases (Bebawi and Neugebohrn 1991; Braun et al. 1991; Cappers, Neef, and Bekker 2009; Cappers, Bekker, and Jans 2012; Neef, Cappers, and Bekker 2012) and was conducted using a binocular microscope with 10x magnification.

Ethnographic analogies are also used throughout this paper to interpret the possible uses of plants identified in the courtyards. These were obtained from the available literature on the flora of Sudan, which also details the uses of plants in contemporary societies (e.g., Harrison and Jackson 1961; Bebawi and Neugebohrn 1991; Braun et al. 1991; Darbyshire et al. 2015). In addition, all ethnographic analogies related to the use of plants in traditional Sudanese medicine were considered. This analysis included specialized references from the Sudan National Centre for Research and other relevant scientific papers on the subject (e.g., El Ghazali 1997; El Ghazali et al. 2008, 2022; Ahmed et al. 2010; Khider 2018; Sakina and Ahmed 2018). In order to further refine these analogies, we carried out a survey in the area of Old Dongola (El Ghaddar village) where we interviewed the local people about the potential uses of the plants in their traditional medicine (Nasreldein et al. 2024; Nasreldein forthcoming). These ethnographic analogies from modern Sudanese societies, which form an interpretive source, were enhanced by a direct historical approach, as these societies are known to be the descendants of the societies of the investigated period. This continuity is observed across language, technologies of food production, and religion (Ḥasan 1973, 142–145; Shinnie 1978, 107; Adams 1986; Ceccarelli-Morolli 1998, 67–72; Kurcz 2007; Żurawski and Łajtar 2014, 84–89; Danys 2022).

### Ground stone tool analysis

A technological analysis was conducted to determine the function of the collected tools. In total, 51 ground stone tools were analyzed. 46 of these objects were gathered from U214, while the remaining five were from U244. The procedure involved morphological description, identification of raw materials, usewear analysis, and facet configuration analyses (Adams 2002, 17–59). The classification of the ground stone assemblage from Old Dongola was conducted by A. Ryś-Jarmużek (forthcoming). The analysis of tools was performed on a macroscopic level to identify the traces of usage. Based on the premises of a techno-functional analysis (Adams 2002; Adams et al. 2006), three primary mechanisms can be identified: abrasive, fatigue, and tribochemical. Each mechanism causes specific types of damage: abrasive wear results in striations, scratches, leveling, and grain edge rounding; fatigue leads to fractures, cracks, and pits; and, tribochemical wear results in polishing or sheening traces (Adams et al. 2006, 46). The three-dimensional position of each tool was measured with a theodolite total station and assigned a Field Number (FN). Subsequently, tools from contemporary contexts were plotted into two-dimensional plans of the courtyards. In order to identify clusters of objects, a heatmap with a radius of 2  m was plotted in QGIS software.

## Results

### Geochemical results

The results obtained from the analysis allowed for observations of variations in the distribution of elements in the courtyard floors (Supplemental Materials 1, 2). The element values in the floor samples were compared against the background level. This comparison revealed significant enrichments and/or depletions of values for several elements, possibly indicating an anthropogenic input.

**Figure 2.**
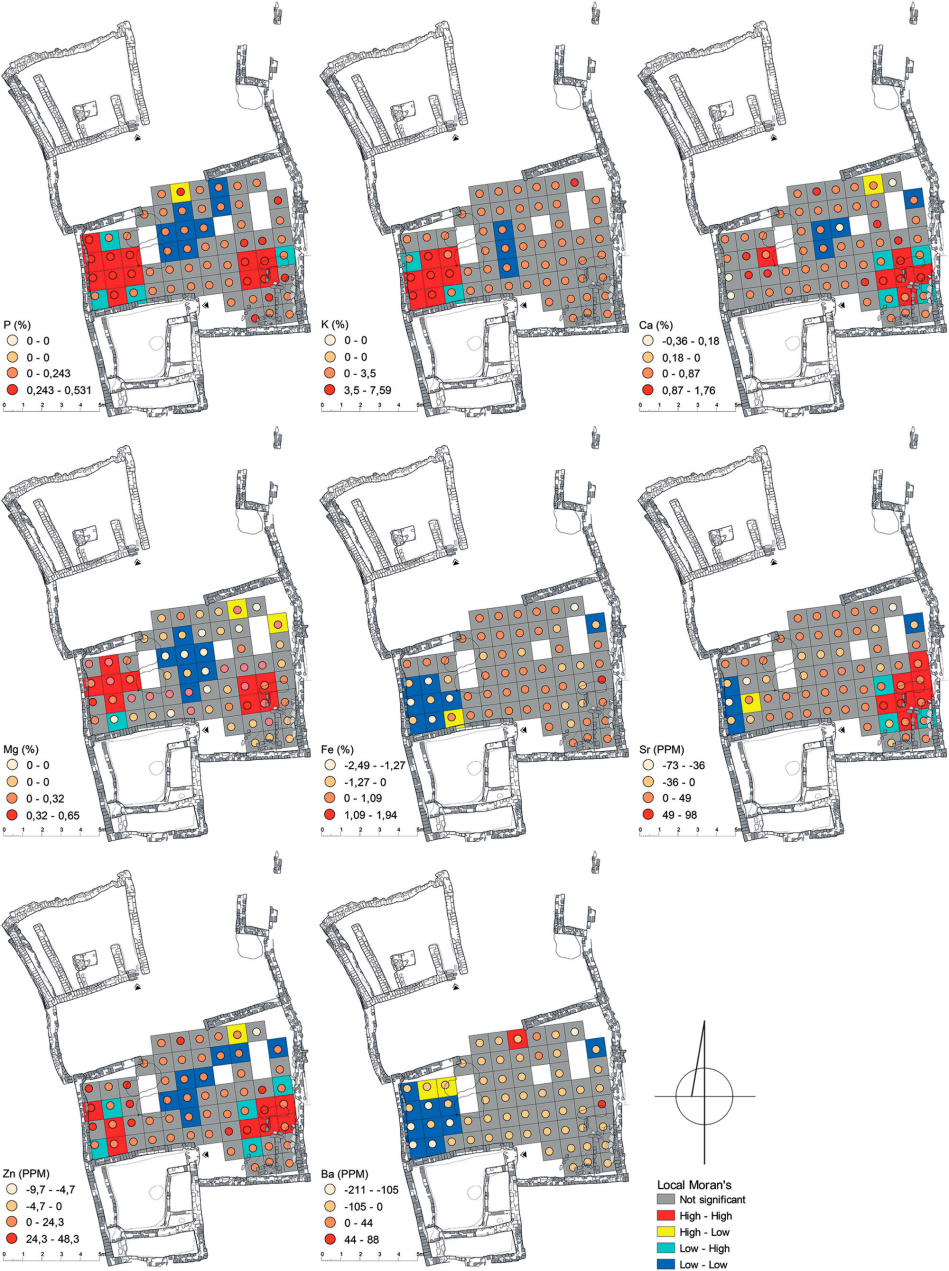
A map displaying element values (P, K, Ca, Mg, Fe, Sr, Zn, and Ba) compared to the background value (mean value of elements of samples of mud brick) and Local Moran’s I calculated for selected elements in Courtyard U214 (drawing A. Wujec).

In Courtyard U214 (Figure 2), the calculation of Local Moran's I for the grid square with element values revealed three areas that contributed the most to a non-random spatial distribution. The first area of elemental enrichment was situated near the western wall of the courtyard, between the southern house wall and a separating wall across the courtyard. This area exhibited an increase in Zn, S, K, Mg, P, and Ca and depletions in Mn, Ba, Zr, Cr, Th, La, Y, Sc, Na, Cu, Al, Ti, Fe, Co, and Ni. The second area, located in the middle part of the courtyard, showed depleted values of Zn, S, K, Mg, P, and Ca. The third area was found in the southeastern corner of the courtyard and displayed elevated levels of Mg, Mn, Ba, P, Cu, Sr, Zn, Na, and Ca.

**Figure 3.**
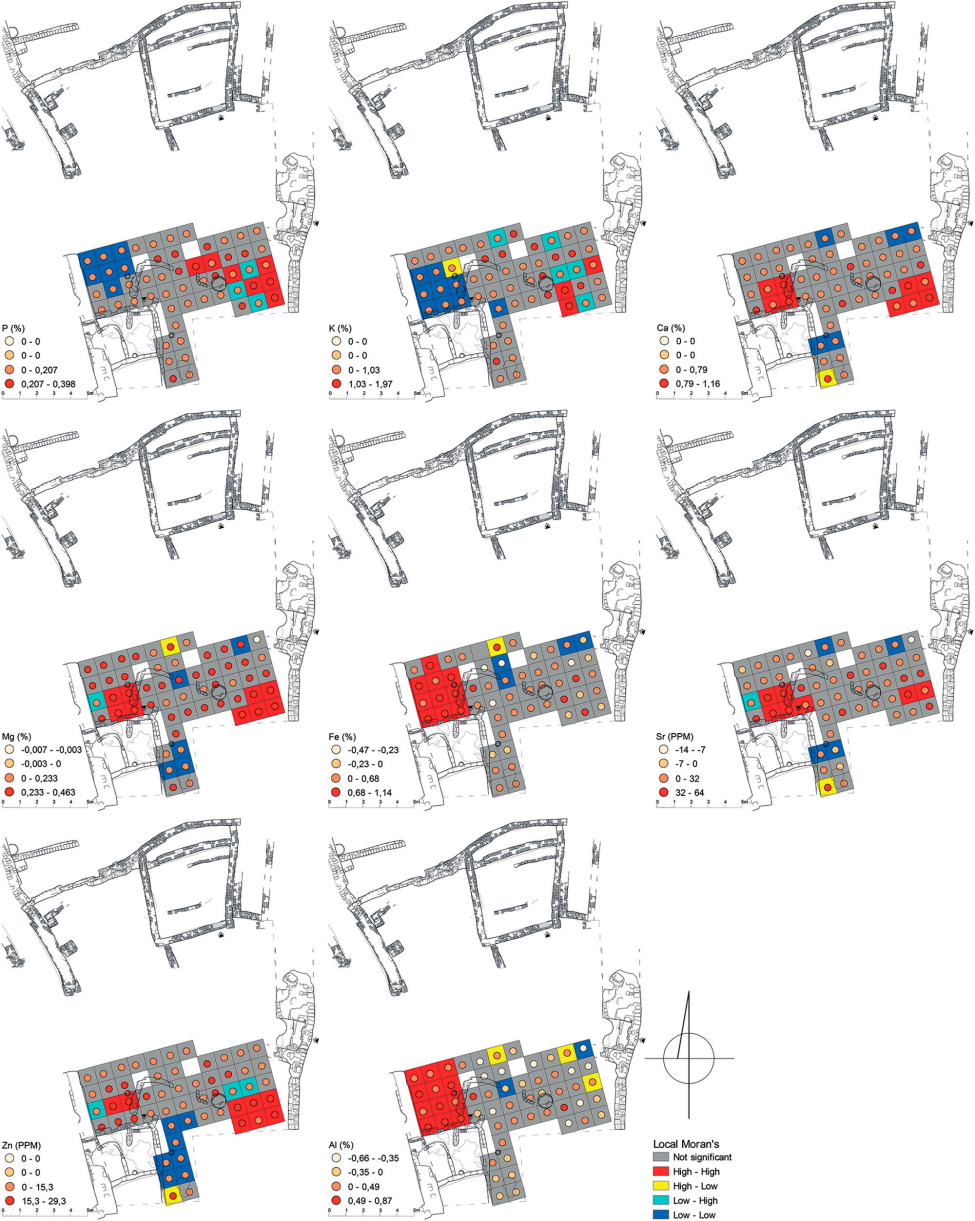
A map displaying element values (P, K, Ca, Mg, Fe, Sr, Zn, and Al) compared to the background value (mean value of elements of samples of mud brick) and Local Moran’s I calculated for selected elements in Courtyard U244 (drawing A. Wujec).

The calculations for Courtyard U244 showed also three distinctive areas (Figure 3). The first area was located by the western wall of the courtyard and had elevated values of Mn, Sr, Mg, Ca, Y, Cu, Ni, Al, Fe, Ti, Co, La, V, and Zr and depleted K and P. The second area, located by the southern edge of the excavated space, was characterized by depletion of Sr, Zn, Cu, La, Ti, Pb, Mg, Co, and Ca. The third area was located in the southeastern corner of the excavated space of the courtyard and had elevated values of Zn, K, P, Na, Sr, Ca, and Mg.

### Ground stone tool analysis results

It was possible to categorize recovered tools into three functional groups: grinding tools, percussive tools, and abrading/polishing tools.

The most prevalent category is grinding tools (n = 28), including five querns and 22 grinders. All tools in this category were crafted from locally accessible sandstone, which is known for its hardness, appropriate texture, and durability. Two levels of grinding activity were observed on the surfaces: leveling of grains and minerals and the presence of scratches and stations (Adams 2002, 98). In certain cases, sheen wear can also be observed on the surfaces. Querns can be divided into two types based on their working surface: two Q1 types with a flat surface (FN968, Inv. No. 6408) and two basin-like Q3 types (FN856, FN843), with one example where it was not possible to determine the exact type (FN844). Based on morphology, the grinders can be categorized into three types—eight spherical examples of G1 type (FN895, FN842, FN964, FN974, FN1064, FN1065, inv. no. 6274, and inv. no 7120) and 12 tools of G2 type that are not as uniform in shape but have larger and heavier flat working surfaces (FN836, FN839, FN977, FN981, FN848, FN972, FN968, FN1054, FN1063, FN1055, FN1061, and Inv. No. 5951), as well as two discoidal-shaped tools of G3 type with a convex surface (FN1056 and Inv. No. 6406). By examining the configuration of working surfaces, some variations in the usage of tools can be identified. Basin-like querns could have been used along with spherical or discoidal tools in a circular or reciprocal motion. In contrast, querns with a flat working surface and grinders were utilized together in a back-and-forth motion, held by one or two hands.

A total of 16 items were identified as percussive tools. This identification is primarily based on the observation of impact fractures, cracks, pits, and flake scars visible on the surfaces of these items (Adams 2002, 151). Out of these 16 items, two are larger and heavier in size. They bear deep impact fractures and flake scars and are classified as hammers (FN964 and FN967). They were more suitable for long-term use and for delivering more forceful strokes. The remaining tools are classified as pounders and were likely used with less force compared to the hammerstones. As a result, the impact fractures on these tools are more uniform. Among the tools, seven have a spherical shape (P1 type) and show pounding marks on all surfaces (FN847, FN982, FN841, FN975, FN973, Inv. No. 6929, and Inv. no 7079). Five tools are classified as P2 type, which is characterized by the location of the working surface on one of the edges (FN1060, FN1066, FN1059, FN1062, and Inv. No. 7082). One tool is classified as a passive tool, specifically an anvil (FN976). FN840 is classified as a scraper due to the presence of a sharp edge.

A total of eight objects were recognized as only raw material, as no usewear was detected on their surfaces. Among these, seven are quartz pebbles (FN835, FN845, FN846, FN980, FN970, FN966, and FN1057) and show traces of breakage. Additionally, one object is a fragment of a sandstone chip (Inv. No. 7086).

The analysis of ground stone tools revealed that they were used for various functions. Grinding was the most common (n = 20), followed by grinding/pounding (n = 6), pounding (n = 13), and crushing (n = 4). In some cases, the tools were used for both grinding and pounding simultaneously, so they were classified as grinders with the function of grinding/pounding. The functions of the tool were associated with particular activities or sets of activities classified into two general groups—subsistence and craft. Whenever possible, a more specific activity, such as food processing, pigment processing, or surface treatment, was established by analyzing traces of residues on the tools’ surfaces or by comparison of wear patterns with ethnoarchaeological sources (Ryś-Jarmużek forthcoming).

**Figure 4.**
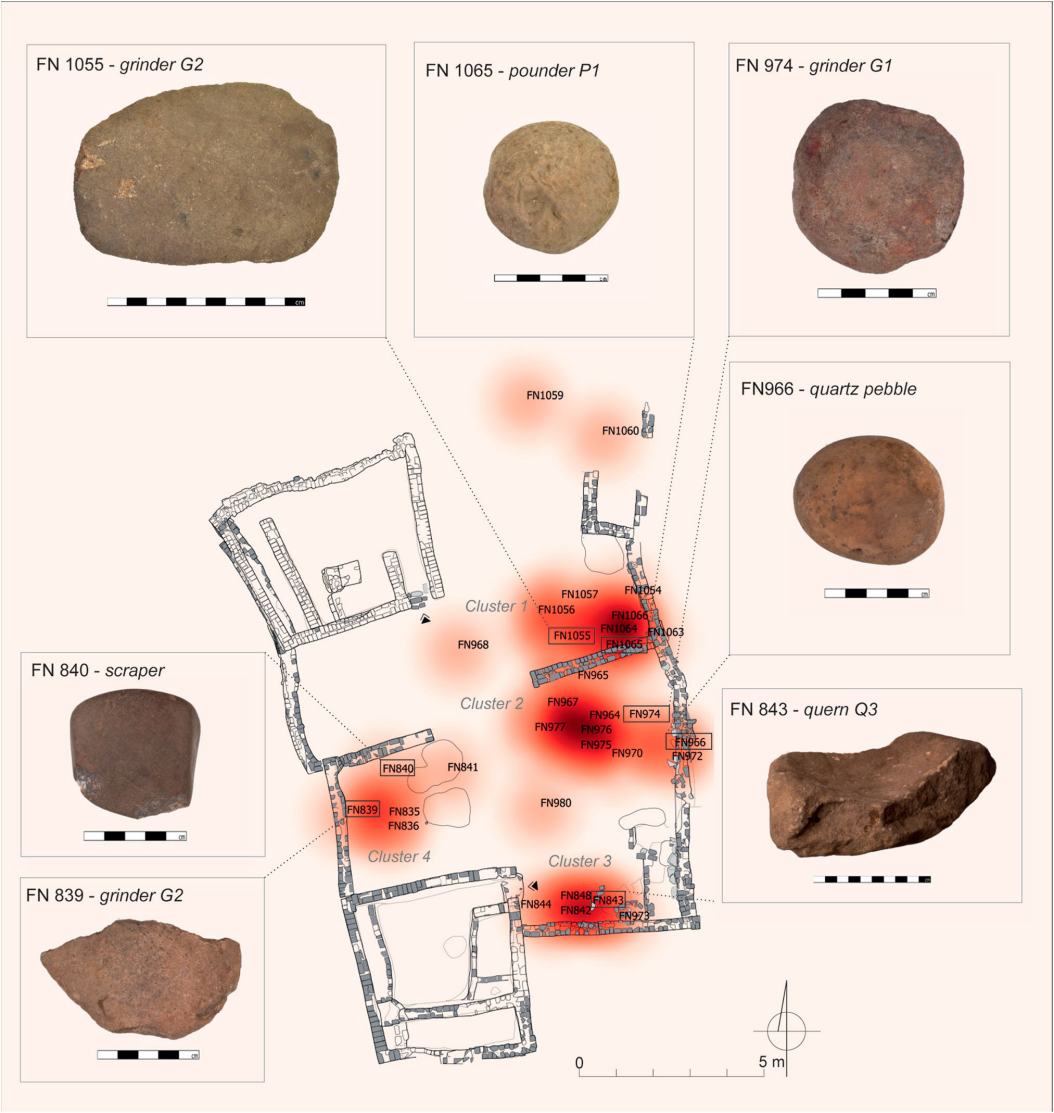
Distribution of ground stone tools with marked clusters (d: 2  m) in Courtyard U214, with examples of tools (drawing by A. Wujec; photo by M. Skarżyński).

In Courtyard U214, four main clusters of tools were observed. Three clusters were located along the eastern wall and numbered from one to three, starting from the north. The fourth cluster was situated by the western wall (Figure 4, Table 3). The assemblage of ground stone tools found in Courtyard U244 (see Table 3) was comprised of only five tools, including only two whose precise location was determined: a grinder G2 (FN1061) and a pounder P2 (FN1062).

**Table 3.** Ground stone tool assemblages according to types, function, and related activity.

Courtyard	Cluster	FN	Type	Subtype	Preservation	Function	Activity
U214	1	1057	raw material		fragment		craft
		1056	grinder	G3	fragment	grinding	subsistence/food processing
		1054	grinder	G2	fragment	grinding	subsistence/food processing
		1063	grinder	G2	fragment	grinding	
		1055	grinder	G2	complete	grinding	subsistence/food processing
		1066	pounder	P2	complete	pounding	
		1065	pounder	P1	complete	pounding	surface treatment/pecking
	2	964	grinder	G1	complete	grinding/pounding	craft/processing
		964	hammer	H	complete	crushing	processing
		975	pounder	P1	complete	pounding	craft/processing
		970	raw material	RM	fragment		craft
		966	raw material	RM	fragment		craft
		972	grinder	G2	complete	grinding	craft/pigment processing
		974	grinder	G1	complete	grinding/pounding	craft/pigment processing
		967	hammer	H	complete	crushing	craft
		976	anvil	An	fragment	pounding	craft
	3	842	grinder	G1	complete	grinding/pounding	craft
		843	quern	Q3	fragment	grinding	subsistence/food processing
		844	quern	Q	fragment	grinding	craft
		848	grinder	G2	complete	grinding	craft/clay processing
		973	pounder	P1	complete	crushing	craft/processing
	4	836	grinder	G2	fragment	grinding	subsistence/food processing
		839	grinder	G2	fragment	grinding	subsistence/food processing
		835	raw material	RM	fragment		craft
		841	pounder	P1	complete	pounding	craft
		840	scraper	P2	complete	scraping	craft/surface treatment
U244		1061	grinder	G2	fragment	grinding	
		1062	pounder	P2	fragment	pounding	

### Archaeobotanical results

The assemblage of archaeobotanical material (Table 4) found in Courtyards U214 and U244 revealed a wide variety of economically important plants, as well as wild plants. The plant remains were exceptionally well-preserved. The majority of samples were preserved by desiccation, and some samples contained a mixture of desiccated and charred plant remains, mostly collected from the midden contexts.

**Table 4.** Total count of the identified plant remains per sample.

Genus/Species	Context 1180 (S.2724)	Context 1185 (S.2743)	Context 1185 (S.2746)	Context 1185 (S.2747)	Context 1185 (S.2787)	Context 1553 (S.2792)	Context 1554 (S.2788)	Context 1556 (S.2790)	Context 1557 (S.2791)	Context 1666 (S.3056)	Context 1666 (S.3057)	Context 1580 (S.2812)	Context 1580 (S.2815)	Context 1580 (S.2816)	Context 1669 (S.3062)	Context 1669 (S.3063)	Context 1669 (S.3064)
*Sorghum bicolor* (L.) Moench	93	15	300	13	44	5	186	6	3	16	133	13	1	220		258	14
*Sorghum halepense* (L.) Pers.	9		15		105		45					2		2			
*Triticum aestivum* L.	2						8	1				9	2				
*Hordeum vulgare* L.	13	4	11		9		165	8	7	8	9	16		7	5	57	5
*Portulaca oleracea* L.	22	23	30	260	100	110	5	8	400	25		7	36	33	14	73	15
*Raphanus sativus* L.	2		5			3	15	7	13		6	18	7		2	5	
*Solanum melongena* L.									1								
*Cucumis melo/sativus*	8	2	4	10	2	30	22			8	5	21	23	6	22	5	2
*Brassica nigra* (L.) W.D. J. Koch		1						2	3							6	
*Carthamus tinctorius* L.												9	2	2			
*Lepidium sativum* L.							5		2	2	2	3		2			
*Lupinus albus* L.											3	15				2	
*Coriandrum sativum* L.							2										
*Citrullus lanatus* (Thunb.)				2		5			11	3	2	6	12				
*Vitis* sp.													1			1	
*Phoenix dactylifera* L.							1										
*Ficus* sp.								1						2			
*Ceratonia siliqua* L.							1										
*Echinochloa* sp*.*	272	168	210	81	145	250	485	8	750	85	66	123	24	366	33	323	53
*Cyperus rotundus* L.	580	195	213	350	165	210	156	68	200	120	110	139	410	234	113	144	53
*Cynodon dactylon* (L.) Pers.		17	203		133	25	210	4	65	15	43	115	67	68	19	55	22
*Glinus lotoides* L.	27	120	60	10	34		66			34	213	17	22		139	184	56
*Coronopus niloticus* (Delile)		15	50	10	10	40	84		5	9	18	42	14	35	73	18	11
*Digitaria* sp.	16	1	23	7	14	1	28		1	5	8	3		2	2	9	
*Setaria* sp.	58	4	4	2	9	8	18		11	2	9	21		12	2	8	4
*Eragrostis* sp.	18	8	2	7	18	12	186		60	10	12	16	12	28		40	6
*Pennisetum* sp.	85	2	9		18		3	8	5		18	3		8		25	
*Dactyloctenium aegyptium* Willd.	1				2	1			2	3	6						
*Crypsis schoenoides* (L.) Lam.	12	12	64	6	5	11	22				5	7	3	1	22	36	2
*Cleome* cf. *gynandra* L.	6	7	7	2		9	11		7	10	5	12	42	4	2	6	
*Citrullus colocynthis* (L.) Schrad.	6	5	1	24	4	20	18			11	55	7	11	2	18	2	2
*Amaranthus* sp.	4		4	11	5	7	5	2	4	12	22	17	23			5	1
*Eclipta prostrata* L.		1				105	4		1		3		3	2		2	
*Solanum nigrum* L.	5		3	3													
*Solanum* sp.						10			1			3	9	1	11	3	
*Verbena supina*	50			2		5	6		2	2		1	22	5	2	4	
*Acacia* sp.							1	1			3	7	1			1	
*Ambrosia* sp.			1						1	2		27	1		2		
*Heliotropium ovalifolium* Forssk.						1			1				17	2		2	
*Heliotropium europaeum* L.				1		1	1	1					2		10	3	
*Silene* sp.	1	1														1	
*Cyperus* sp.	36		48		30												
*Nauclea latifolia* Sm.		1															
*Boerhavia* sp.													1			1	
*Fimbristylis bisumbellata* (Forssk.) Bubani					3												
*Senna* sp.																	
*Rumex* sp.				1									1	1			
*Hyoscyamus muticus* L.		1	1							2							

**Figure 5.**
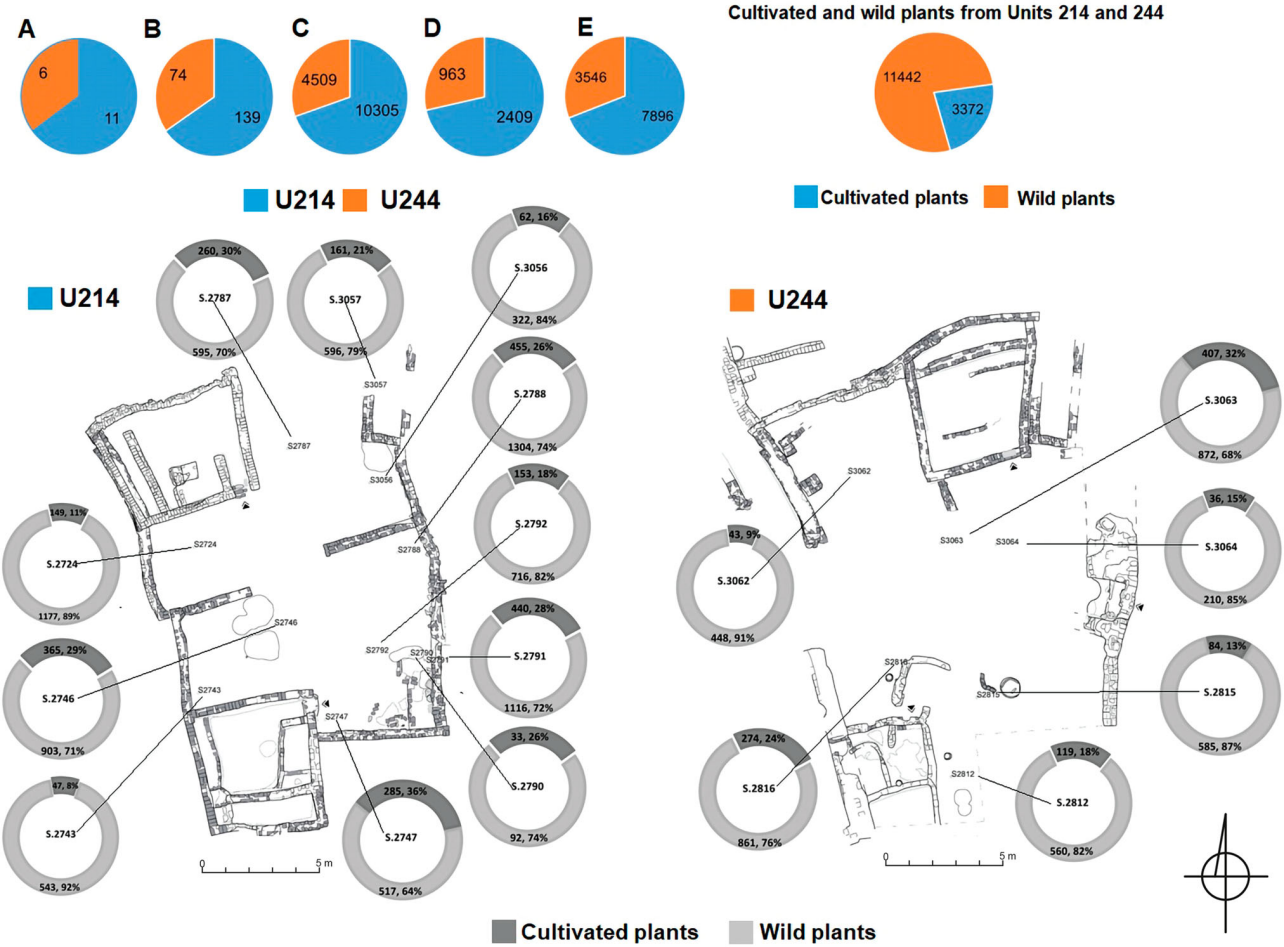
Distribution of the archaeobotanical remains in U214 and U244. The pie charts on the left top indicate: A) total samples, B) sample volumes (L), C) total number of plant remains, D) total counts of cultivated plants, and E) total counts of wild plants. Black numbers in pie charts represent seed counts and their percentages (drawing by A. Wujec and M. Nasreldein).

A total of 213 L of archaeological deposits were collected as samples from both units, with 139 L from U214 and 74 L from U244. The sampling yielded 14,814 seed remains, with 10,305 from U214 and 4509 from U244. It is worth noting that, out of the entire assemblage, only 3372 seed remains were identified as cultivated plants, making up only 23% of the total amount. The remaining 77% (11,442 seed remains) consisted of wild plants, which dominated the assemblage. The majority of cultivated plants were found in U214, with 2409 (71%) compared to only 963 (29%) in U244 (Figure 5). The archaeobotanical assemblage from all sediment categories provided us with comparable results regarding the distribution of plants throughout the courtyards. The samples varied in terms of the total number of seed remains and, to a lesser extent, the presence of certain species (Supplemental Material 3).

Three different types of occupational deposits were observed in the courtyards. The first type consisted of a sandy layer with a significant amount of organic material, including animal dung, which covers most of the area in both courtyards. From this category, seven sediment samples were collected from U214 and six samples from U244. The second type includes ashes and burnt organic matter from hearths. Only one sediment sample represents this category and was collected from U214 S.2790 (see Table 4). The third type consists of tamped animal dung and is represented by three samples from U214, specifically S.2724, S.2788, and S.2791 (see Table 4).

## Discussion

### Courtyard U214

#### Geochemistry

**Figure 6.**
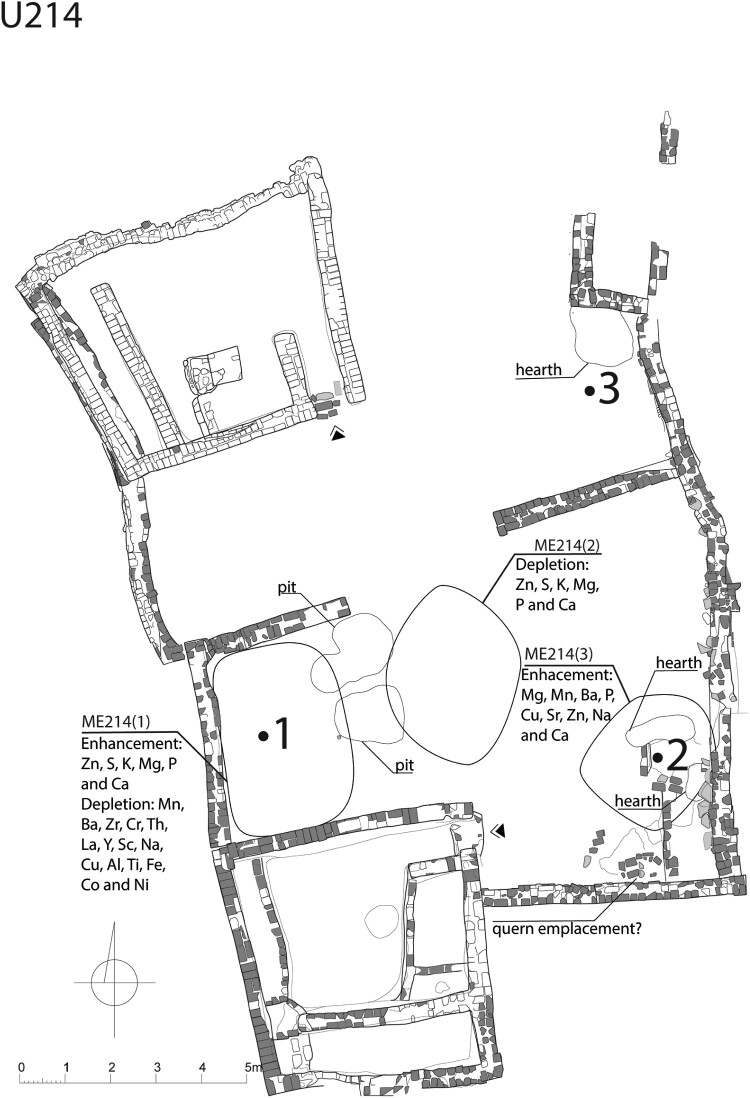
Areas of elemental enrichment/depletion and activity areas (indicated by Arabic numerals) in Courtyard U214 (drawing by A. Wujec).

Three areas, ME214(1), (2), and (3) (Figure 6), can be identified by their distinctive elemental characteristics associated with domestic functions. The interpretation of these areas remains ambiguous due to a wide range of activities that are linked to similar elemental input (Schiegl et al. 1994; Wells 2004; Tsartsidou et al. 2007; King 2008; Oonk, Slomp, and Huisman 2009; Middleton et al. 2010; Wyżgoł and Woronko 2024).

The elemental composition of two areas with significant enrichments can be connected to similar activities (see Figure 6, Table 5). However, they differ in terms of how well they match the elemental footprints of these activities. In ME214(1), high levels of P, K, and Zn, which are associated with animal metabolism, are observed, while ME214(3) lacks elevated levels of K. Both areas exhibit elemental enrichments related to plant ashes, but ME214(1) additionally shows depleted values of several metallic elements. This aligns with the locally observed pattern, suggesting that, compared to the Nile silt background, ashes have the lowest content of most analyzed elements (Wyżgoł and Woronko 2024). Additionally, the presence of S enrichments may indicate the presence of anhydrite, a major component of *Tamarix sp.* ashes (Tsartsidou et al. 2007, 1264). Furthermore, both areas show enrichments in elements typically associated with plant and animal tissues, but ME214(3) aligns more closely with the pattern of enrichments related to the presence of bone remains. This qualitative comparison suggests that ME214(1) was likely used for the deposition of middens, including ashes, and possibly for keeping livestock. This may be supported by the presence of a nearby rubbish pit. In contrast, ME214(3) was most likely a food preparation area, which is corroborated by the proximity of two hearths and a presumed quern emplacement. ME214(2) (see Figure 6), characterized by depleted elements related to human activities, suggests that the central part of the courtyard was not used for domestic tasks and was primarily used for communication purposes.

**Table 5.** High values of elements within the enrichment areas and related residues and activities in U214.

Courtyard	Area	Activity/function	Residues (elemental input)
U214	ME214(1)	middens	ashes (Mg, P, K, S); plant tissues (P, K, Ca)
		livestock presence	feces (P, K); urine (P, Zn); plant tissues (P, K, Ca)
	ME214(3)	food preparation	bones (P, Ba, Ca, Sr); plant tissues (P, Ca, Sr); animal tissues (P); ashes (Na, Ba, P)
		middens	ashes (Na, Ba, P); plant tissues (P, Ca, Sr)
		livestock presence	feces (P); urine (P, Zn); plant tissues (P, Ca, Sr)

#### Ground stone tools distribution

Four clusters of ground stone tools were found in Courtyard U214 (see Figure 4, Table 3). Cluster 214.1 encompasses various objects that are either broken or worn out. Their variability precludes identification with any specific activity; therefore, it is plausible that this location served as either a midden or an area for storing broken tools to be repurposed in the future. Cluster 214.2 consists of tools associated with crafts (see Table 3), specifically the processing of materials like pigment or quartz pebbles, as indicated by the presence of raw materials. Cluster 214.3, located next to a structure interpreted as a quern emplacement, consisted of five tools used in food processing. These findings support the identification of this space as a cereal processing station. The assemblage of Cluster 214.4 is not homogeneous in terms of function, and the tools show clear signs of wear and tear. This suggests that they may have been discarded in this area when they were no longer useful, so Cluster 214.4 bears some similarities to Cluster 214.1.

#### Archaeobotany

The plant assemblage from U214 (see Table 4) was comprised of a wide range of cultivated and wild plant species, predominantly consisting of weedy plants. In a domestic setting, these plants could have been used as animal fodder or as fuel for domestic hearths. The distribution of the botanical remains in U214 (see Figure 5) allowed for the identification of specific locations where plant processing or consumption took place.

The presence of wild weed plants, particularly a significant amount of *Glinus lotoides* L., in S.2743 indicates the presence of livestock in the nearby area. This plant is commonly used as animal fodder, especially for sheep and goats (El Ghazali et al. 2008; Subramani 2015; Teshome and Feyissa 2015; Saleem 2022). Additionally, the occurrence of plants from various taxa in small quantities, such as a single seed of the African peach fruit (*Nauclea latifolia*), False daisy flower (*Eclipta prostrata* L.), Egyptian henbane (*Hyoscyamus muticus* L.), and Black mustard (*Brassica nigra*), may suggest the disposal of waste or by-products from domestic activities.

The majority of plant remains found in S.2746 consisted of sorghum in the form of spikelets and rachis, which indicate processing of cereals, e.g., dehusking. The samples collected from the area adjacent to two hearths and a quern emplacement in the southeastern corner came from different archaeological contexts, including the fill of a hearth containing ashes and burnt organic matter (S.2790), tamped animal dung (S.2791), and a sandy occupational layer (S.2792). These assemblages consisted predominately of a wide variety of wild weed plants, making up over 70% of each sample. These wild plants were likely used as fuel or were associated with activities such as grain processing, including dehusking, possibly being accidentally brought from the fields alongside the cereals. Despite this, the area was also used for food preparation. The processing of cereals was evident from the presence of sorghum, bread wheat, and hulled barley remains in sample S.2790. Additionally, other economically important plant remains were discovered, such as seed remains of radish and eggplant in S.2791.

In S.2792, a notable discovery was the presence of a substantial quantity of *Eclipta prostrata* L. seeds, commonly referred to as false daisy flowers. While the significance of this finding may not be immediately apparent, it becomes more meaningful when considering the plant’s historical and cultural importance. *Eclipta prostrata* L. has long been utilized in traditional medicine practices, particularly in the preparation of hair oils and the extraction of hair dye (e.g., Feng et al. 2019; Timalsina and Devkota 2021; Silalahi 2022; Yang et al. 2023). Understanding the historical context of this plant sheds light on its potential use within ancient societies and highlights the interactions between botanical resources and human culture. This may suggest that this area was used for activities other than cooking. S.2790, collected from a hearth, contained fully charred seed remains, primarily from wild plants, which may indicate their use as fuel through their presence in animal dung.

Sample S.3056 was primarily comprised of wild plants, but it also contained a significant quantity of sorghum spikelets and hulled barley rachis, indicating the presence of cereal processing, including dehusking. Furthermore, various other remains related to food, such as cress and watermelon, were discovered. It is worth noting that the majority of the collected seed remains from this sample were fully charred, suggesting activities related to food preparation.

In S.3057, a significant quantity of colocynth seeds was discovered, preserved by charring, suggesting deliberate practice. This is notable due to the local community’s traditional utilization of colocynth seeds in various folkloric practices (Osborn 1968; Bebawi and Neugebohrn 1991). Specifically, these seeds are integral to the production of *tar*, a substance deeply rooted in local knowledge. Tar serves multiple purposes within the community, including the treatment of mange in camels and the tanning of animal skins for crafting water containers (see Osborn 1968; Bebawi and Neugebohrn 1991; Badura 2012; Wolcott et al. 2021).

#### Activity patterns

Through geochemistry, artifact distribution, and archaeobotany, three activities areas were identified. Two of these areas were found within the space where the chemical analysis was carried out, while one was located outside of it. The first activity area (Table 6) was situated near the western wall of the courtyard, close to two pits. The chemical input ME214(1) can be associated with the disposal of rubbish. Cluster 214.1 contained various types of tools that were either broken or worn out. This implies that they were discarded when they were no longer useful, rather than being connected to any domestic production performed in this spot. The plant remains found in sample S.2743 also support this interpretation, as they include a variety of different seeds from different plants, but in small quantities.

**Table 6.** Spatial relations of areas of elemental enrichment/depletion, tool clusters, and botanical samples in U214.

Activity area	Elemental enrichment/depletion	Tools	Botanical sample
First	ME214(1)	Cluster 214.4	S.2743
	ME214(2)	Cluster 214.2	S.2746
Second	ME214(3)	Cluster 214.2	S.2790 (hearth)
		Cluster 214.3	S.2791
			S.2792
Third		Cluster 214.1	S.3056
		Cluster 214.2	S.2788
		Cluster 214.3	S.2747
			S.2724
			S.2787
			S.3057

The second activity area (see Table 6) was located in the southeastern part of the courtyard, near two hearths and a presumed quern emplacement. The elemental composition of ME214(3) ambiguously indicates activities related to food processing, as well as keeping livestock. Additionally, the tools found in two neighboring clusters, 214.2 and 214.3, were also associated with food and possibly with mineral processing. Given the presumed discard pattern (Murray 1980, 497; Schmader and Graham 2015, 30–31), these clusters should be, in fact, associated with the neighboring second activity area. Furthermore, three botanical samples (S.2790, S.2791, and S.2792) contain cereals such as sorghum, bread wheat, and hulled barley, as well as a large number of wild plants. This suggests that grain processing and cooking took place in this area and that livestock was present.

The third activity area (see Table 6) was most likely situated in the northeastern part, outside of the area of elemental analysis. It was near a cluster of ground stone tools and a hearth. Based on the botanical remains found in this area (S.3056), it is assumed that it was also used for food processing and discarding waste products. This is supported by the remains of edible plants, including sorghum, hulled barley, cress, and watermelon.

### Courtyard U244

#### Geochemistry

**Figure 7.**
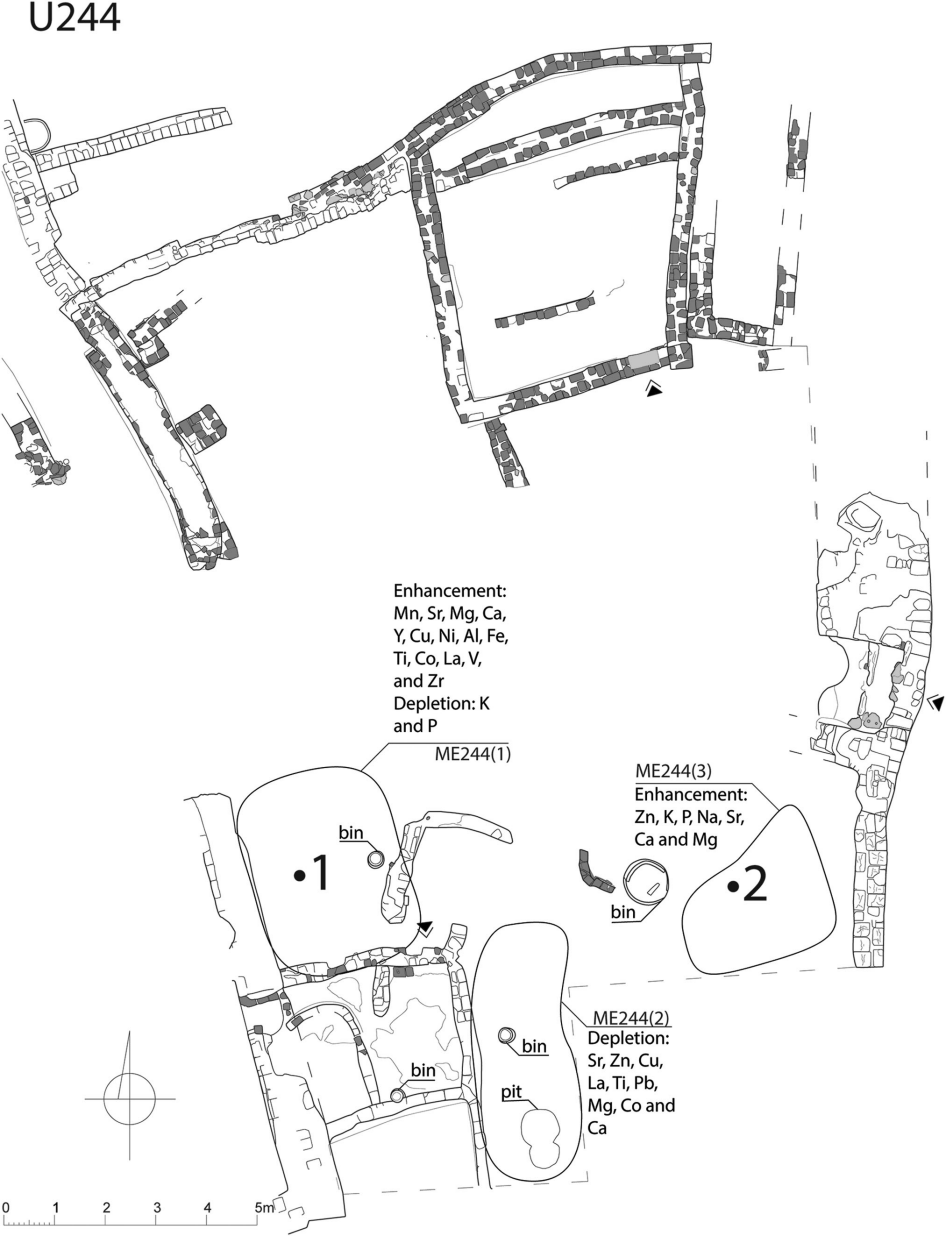
Areas of elemental enrichment/depletion and activity areas (indicated by Arabic numerals) in Courtyard U244 (drawing A. Wujec).

In Courtyard U244, three distinct areas were identified (Figure 7, Table 7), which were characterized by either the enrichment or depletion of certain elements associated with human activities (Schiegl et al. 1994; Wells 2004; Tsartsidou et al. 2007; King 2008; Oonk, Slomp, and Huisman 2009; Middleton et al. 2010; Wyżgoł and Woronko 2024). In ME244(1), the presence of ashes and plant tissues can be associated with the presence of certain elements; however, the significant depletion of P and K contradicts the idea that these elements are related to the processing of plant and animal tissues. On the other hand, the presence of enrichments of metallic elements such as Fe or Ti suggests a possible association with craft activities, such as pigment processing. Conversely, the depletion of P and the enrichments of Al, Fe, Ti, and Mn may have originated from eroded Nile silt from a large sun-dried brick wall. ME244(3) exhibited a strong correlation with patterns of enrichment that are typically related to food preparation, particularly the processing of plant and animal tissues, as well as the presence of animals. Furthermore, this area was in close proximity to a large storage bin. The depletions in metallic elements observed in ME244(2) (see Figure 7) match elemental patterns associated with human activities. One possible explanation could be variations in the mineral composition of the background material (see Wyżgoł and Woronko 2024).

**Table 7.** High values of elements within the enrichment areas and related residues and activities in U244.

Courtyard	Area	Activity/function	Residues (elemental input)
U244	ME244(1)	middens	ashes (Mg, Ca); plant tissues (Sr, Ca)
		craft	pigments (Fe)
	ME244(3)	middens	ashes (Mg, P, K); plant tissues (P, K, Ca, Sr)
		food preparation	plant tissues (P, K, Ca, Sr); animal tissues (P); bones (Sr, Ca, P, K)
		livestock presence	feces (P, K); urine (P, Zn); plant tissues (P, K, Ca, Sr)

#### Ground stone tools distribution

The assemblage of ground stone tools found in Courtyard U244 (see Table 3) was comprised of only five tools, which prevented cluster analysis.

#### Archaeobotany

The archaeobotanical assemblage from U244 (see Figure 5, Table 4) indicated a diverse range of cultivated and wild plant species, primarily consisting of wild weed plants that were likely used as animal fodder or as fuel in domestic hearths. The plant assemblage yielded similar results as U214 in terms of plant by-products and remnants of cereal processing, specifically sorghum and hulled barley. Remnants of discarded food plants, such as purslane, cucumber, and watermelon, as well as other weed plants like *Echinochloa* sp., *Cyperus rotundus*, *Cynodon dactylon*, and *Coronopus* cf*. niloticus*, known for their use as animal fodder (Harrison and Jackson 1961; Bebawi and Neugebohrn 1991; Braun et al. 1991; El Ghazali et al. 2022), were discovered in varying proportions in each sample from this unit. This suggests that the courtyard was the site of activities related to animal husbandry and/or the disposal of plant by-products.

The plant assemblages from S.2816 and S.3063 yielded the highest number of sorghum and hulled barley rachis, along with a single grape seed, indicating food processing. S.2815 provided a wide variety of economic crops, including watermelon, cucumber, purslane, radish, and grape seeds. These plants confirm the assumed food production in this area. Additionally, botanical remains of safflower (*Carthamus tinctorius* L.) were also identified, which was probably used as a dye plant in textile production (Zohary and Hopf 2000; Zohary, Weiss, and Hopf 2012; Fuller 2020). The evidence of safflower seeds suggest that it was probably grown for its oily seeds, which can be eaten roasted or pressed for oil, and it is also used as a substitute for saffron, as previously discussed by Fuller and Edwards (2001). Thus, the discovery of its remains in this courtyard suggests an interpretation of disposal of plant by-products.

In addition, the plant assemblages from U244 presented a diverse range of plants utilized in traditional medicinal practices, for example in S.2812, S.2815, and S.2816 (see Figure 5, Table 4). Plants falling into this classification were represented by the remains of seeds from *Citrullus colocynthis* (L.) Schrad., *Amaranthus* sp*.*, *Eclipta prostrata* L., *Solanum nigrum* L., *Ambrosia* sp., *Acacia* sp., and *Hyoscyamus muticus* L. (see Harrison and Jackson 1961; Bebawi and Neugebohrn 1991; Braun et al. 1991; El Ghazali et al. 2008, 2022; Ahmed et al. 2010; Sakina and Ahmed 2018).

#### Activity patterns

Two potential activity areas were identified within the area of the elemental analysis. Given the scarcity of tools found in this courtyard, the interpretation was based on the elemental composition and archaeobotanical samples. The first activity area (Table 8) was situated next to the western wall. It exhibited high levels of elements that likely originated from craft or building materials. This suggests that it may in fact not have been associated with domestic tasks. However, the large quantity of sorghum spikelets and rachis in S.2816, along with a significant presence of weed plants, indicates the processing of grains here, including dehusking.

**Table 8.** Spatial relations of areas of elemental enrichment/depletion and botanical samples in U244.

Activity area	Elemental enrichment/depletion	Botanical sample
First	ME244(1)	S.2816
	ME244(2)	S.2812
Second	ME244(3)	S.2815
		S.3062
		S.3063
		S.3064

The second activity area (see Table 8) in the southeastern corner was linked to food processing and the presence of livestock, based on its elemental composition. Both activities were evident in the composition of the botanical assemblages from a neighboring sample, S.2815, which contained remains of edible and wild plants, constituting 87% of the total number of identified remains, used as animal fodder or fuel.

### Relations between the types of residues

The datasets presented here provide evidence to complement each other and give insights into various aspects of courtyard functions and post-depositional processes. The elemental composition identified may reflect domestic activities or signal past anthropogenic input. Although the statistically significant clusters of high/low values should, as suggested earlier, correspond to the actual locations of activities, given presumed post-occupational processes (see Wyżgoł and Woronko 2024), their identification with particular activities remains ambiguous. This ambiguity may be due to the occurrence of overlapping activities, such as food preparation and the presence of livestock, which could have changed seasonally or daily. The clusters of tools identified in U214, associated with particular activities, occurred at the edges of areas of elemental enrichments and helped identify two types of middens. The first pattern aligns with known ethnographic models (Murray 1980, 497; Schmader and Graham 2015, 30–31) which show that tools are typically put away at the end of an activity or day in regularly cleared areas and are often later found lying along courtyard walls. Therefore, areas on the edges of activity areas should be identified as waste disposal zones, consisting of light refuse and items set aside for future use. The second type, comprising decomposing plant and animal fragments, as well as ashes contributing to elemental input, were likely located farther away from the workspace, such as in the first activity area in U214. Finally, the well-preserved botanical remains support the identification of activities based on other types of residues both in U214 and U244, although the composition of samples may suggest that domestic activities also occurred in the space without elemental enrichments. Such is the case of U244 and Sample S.2812, including crops such as sorghum, hulled barley, purslane, and cucumber, as well as some rare finds like radish seeds, garden cress, lupin, and safflower in large numbers. This may also indicate that botanical remains were horizontally mobile residues, especially given the weather conditions in Old Dongola, and were deposited far from their original location. Alternatively, this pattern of deposition for botanical remains may indicate short-term activities that did not leave distinguishable elemental traces nor require durable stone tools. The co-occurrence of cereals and other edible plants with low levels of anthropogenic elements may also be attributed to the storage of staples in clay bins, which may not leave detectable elemental traces, unlike the processing of plant/animal tissues, burning, or animal presence.

### Courtyards as intensive spaces

In both courtyards, only activities directly related to household subsistence were detected. The dwellers of the house compound U207/210/211/214/225 tended to do food processing in two areas on the eastern wall of the compound, northeast from House U207/225 and northeast from House U210/211. They also disposed of rubbish, most likely from these food preparation areas, within the compound by its western wall, north of House U210/211. In the southern part of the courtyard, the elemental input suggests the presence of livestock in both the western and eastern parts. However, the presence of botanical remains and a layer of animal dung throughout the courtyard suggests that animals freely roamed the entire space. Furthermore, the depletion of elements associated with human activities in the central part of the courtyard indicates that it was primarily used as a walking space. In the uncovered part of the compound’s U242/243/244/246/259 courtyard, U244 lacked features such as a hearth or quern emplacement. This suggests that such features must have existed in the unexcavated area of the courtyard, as cooking is an inherent part of household life. Nevertheless, the elemental composition provides evidence of food preparation in the southwestern part near a clay bin. Additionally, two more bins were found in the southwestern and the southernmost parts of the excavated area, connecting the entire southern portion to domestic work by the dwellers of House U246/259.

Both courtyards demonstrate that the location of tasks was not determined by architectural boundaries or fixed installations. Instead, these spaces should be understood as fluid, like other urban open spaces (see Smith 2008), emerging through the actions performed, rather than as predetermined spaces with fixed functions (see Frichot, Gabrielsson, and Metzger 2016). The repetition of actions created the spaces in both courtyards, rather than this space being predefined by their constructors as the reproduction of an architectural type. Furthermore, the assumed multifunctionality of the whole space of Dongolese courtyards appears to be a generality conceived of by researchers, rather than an actual feature assigned to these spaces by the city’s dwellers (see DeLanda 2016, 72).

## Conclusion

This paper discussed the functioning of space in domestic courtyards based on three datasets—elemental composition, botanical remains, and localization of ground stone tools—in the environmental and geological setting of the Middle Nile Valley. The examination of the elemental makeup of the floors demonstrated the existence of statistically significant clusters of high and/or low values of elements. Differentiations between the compositions of these groups allowed for the identification of domestic activities such as keeping livestock, processing food, burning plants, or depositing waste. Simultaneously, the functional analysis of the ground stone tools indicated that the tools discovered in the courtyards, often multifunctional or changing their purpose over time, could have been used in a variety of activities related to subsistence, such as food preparation, as well as activities related to small-scale crafts. An examination of the spatial distribution revealed that discarded tools tended to cluster. In only one instance were clustered tools connected to a specific activity. In other cases, the clusters were associated with the locations where tools were left behind or as places where they were set aside for future use. Botanical remains provided additional evidence of food preparation and the presence of livestock, as well as other domestic activities such as dyeing or medical practices. However, the identification of specific areas of these activities was impeded by the similar composition of the collected samples.

The way the discussed sets of data were related to each other was primarily determined by the patterns of deposition and the way space was maintained. The presence of installations like quern emplacements or hearths often helped to identify the central part of an activity area. In cases where there were no installations, the only way to determine the location was through the geochemical imprint. Additionally, clusters of discarded tools resulting from regular clearing of the activity areas were found on the edges of elemental enrichments. However, analyzing the functions of tools as clusters did support the otherwise vague identification of the function of the activity areas based on geochemistry. Similarly, botanical remains also helped identify the range of activities, but in all likelihood, their material properties made them susceptible to horizontal movement on the surface, e.g., while exposed to the wind, before their final deposition. This is likely the reason why the indications of archaeobotany and geochemistry do not match in many cases.

The high-resolution methods discussed in this paper can provide sufficient data to reveal complex spatial arrangements and offer a detailed perspective on a space defined by household tasks such as food processing, crafts, or deposition of middens. Instead of labeling this space as simply multifunctional, which implies a stable feature defined in extensive terms, we propose that the courtyards should be defined by the repetition of multiple actions in this dynamic and ever-changing space. Additionally, the use of high-resolution methods can steer research towards the heterogeneity of courtyard houses. Such diversification of formally similar spaces is especially important in many African environmental and social settings (Prussin 1974; Steyn 2005; Fleisher and Wynne-Jones 2012), including the Middle Nile Valley (Lee 1967; Wyżgoł and Deptuła 2023), where house courtyards accommodate various functions and have a high potential for the creation of relationships.

## Supplementary Material

Wyzgol_SM3_RFP.xlsx

Wyzgol_SM2_RFP.xlsx

Wyzgol_SM1_RFP.xlsx
